# Takotsubo Syndrome: Clinical and Diagnostic Characteristics of 26 Consecutively Hospitalized Patients Over a Five-Year Period

**DOI:** 10.14740/cr2249

**Published:** 2026-08-31

**Authors:** Mariya Radkova, Danail Rangelov, Kiril Karamfilov

**Affiliations:** a Clinic of Cardiology, University Hospital “Aleksandrovska”, Sofia, Bulgaria

**Keywords:** Takotsubo syndrome, Stress cardiomyopathy, Apical ballooning, Ejection fraction, NT-proBNP, Case series, Outcomes

## Abstract

**Background:**

Takotsubo syndrome (TTS) is a transient form of acute heart failure characterized by reversible left ventricular (LV) dysfunction, predominantly affecting postmenopausal women following emotional or physical stress. Despite its historically benign reputation, in-hospital complications and mortality remain clinically significant. Data from Central and Eastern European populations are underrepresented in large international registries. The study aimed to describe the clinical, echocardiographic, and biochemical characteristics of a consecutive cohort of 26 patients hospitalized with TTS over a 5-year period and to compare findings with published international registry data.

**Methods:**

This is a retrospective observational study (case series) of 26 consecutive patients. Diagnosis was established per Mayo Clinic criteria (2008). Descriptive statistics and Pearson correlation analysis were applied.

**Results:**

The cohort comprised 23 women (88.5%) and three men (11.5%), with a mean age of 71.4 ± 12.0 years. A triggering event was identified in all 26 patients (100%). Mean initial left ventricular ejection fraction (LVEF) was 41.8±11.4%; classic apical ballooning was present in 100%. N-terminal pro-B-type natriuretic peptide (NT-proBNP) showed a moderate inverse correlation with LVEF (ρ = −0.53, Spearman). Mean length of hospital stay was 12.1 ± 10.2 days (median 7). In-hospital complications occurred in 25% of survivors; in-hospital mortality was 7.7% (2/26).

**Conclusions:**

This study is consistent with the predominance of TTS in elderly women with universal triggering events and significant systolic dysfunction at presentation. NT-proBNP showed an inverse correlation with LVEF, reflecting neurohumoral activation proportional to hemodynamic impairment. Mortality and complication rates were at the upper limit of published values. These hypothesis-generating findings support the need for prospective multicenter validation.

## Introduction

Takotsubo syndrome (TTS), also known as stress cardiomyopathy or apical ballooning syndrome, is characterized by transient reversible left ventricular (LV) dysfunction in the absence of obstructive coronary artery disease (CAD), typically precipitated by physical or emotional stress [1, 2]. The condition derives its name from the Japanese term for an octopus trap, reflecting the characteristic systolic apical ballooning first described by Sato et al in 1990 [3]. The diagnosis is established using the Mayo Clinic criteria (2008), which require: (1) transient LV wall motion abnormality extending beyond a single epicardial vascular territory; (2) absence of obstructive CAD or angiographic evidence of acute plaque rupture; (3) new electrocardiogram (ECG) abnormalities or modest troponin elevation; and (4) exclusion of pheochromocytoma and myocarditis [4]. TTS is clinically indistinguishable from acute coronary syndrome (ACS) on presentation, representing 1–3% of all suspected ACS cases [5].

TTS predominantly affects postmenopausal women, who account for 85–90% of reported cases, with a mean age of presentation of 65–70 years [6]. Postmenopausal estrogen deficiency is thought to impair microvascular and endothelial protective mechanisms, increasing myocardial sensitivity to catecholamine excess—the central pathophysiological mechanism. The global incidence of TTS has increased in recent decades, partly attributable to improved diagnostic awareness and the psychological burden of contemporary stressors [7], including the COVID-19 pandemic [8, 9]. Data from the International Takotsubo Registry (InterTAK), the largest global TTS registry enrolling 3,957 patients between 2004 and 2021, show a significant demographic transition: male patients have increased from 10% to 15% (P = 0.003), physical triggers from 39% to 58% (P < 0.001), and short-term mortality has risen [10].

Despite its initial characterization as a benign, self-limiting disorder, TTS carries a non-negligible risk of serious in-hospital complications, including cardiogenic shock, malignant arrhythmias, LV thrombus formation, and death, with rates comparable to ACS [6, 11]. Independent predictors of adverse in-hospital outcomes include physical triggering events, acute neurological or psychiatric disease, high admission troponin, and low left ventricular ejection fraction (LVEF) [6]. Data from the German Italian Spanish Takotsubo (GEIST) Registry have confirmed that older age (≥ 75 years) and LVEF ≤ 35% are independent predictors of in-hospital mortality [12]. There are no prospective randomized data guiding TTS-specific pharmacotherapy; management is therefore based on clinical consensus [13, 14].

The aim of this study is to describe the clinical and diagnostic characteristics, echocardiographic findings, biomarker profile, treatment patterns, and in-hospital outcomes of a consecutive cohort of 26 patients hospitalized with TTS over a 5-year period at a single center, and to compare findings with international registry data.

## Materials and Methods

### Study design and population

This is a retrospective, descriptive, single-center study analyzing 26 consecutive patients hospitalized with TTS. Diagnosis was established according to the updated Mayo Clinic criteria (2008). Patients with insufficient documentation (< 50% of analyzed parameters recorded) were excluded.

### IRB approval

This study was approved by the Ethics Committee of University Hospital “Aleksandrovska”, Sofia, Bulgaria (Protocol No. 4/20.03.2026).

### Ethical compliance

This study was conducted in compliance with the ethical standards of the responsible institution on human subjects as well as with the Helsinki Declaration.

### Data collection

Data were collected retrospectively from medical records using a standardized coding table including: demographics; clinical presentation (symptoms, New York Heart Association (NYHA)/Killip class, vital signs); triggering event (emotional vs. physical/somatic); ECG findings; echocardiographic parameters (LVEF, LV end-diastolic volume (LVEDV), LV end-systolic volume (LVESV), pulmonary arterial hypertension (PAH), valvular regurgitation, LV outflow tract (LVOT) gradient); laboratory values (high-sensitivity troponin T (hs-TnT), creatine phosphokinase (CPK), CPK-MB, N-terminal pro-B-type natriuretic peptide (NT-proBNP), C-reactive protein (CRP), renal function, hematology, electrolytes); in-hospital treatment; length of stay (LOS); complications; and in-hospital mortality.

### Statistical analysis

Statistical analysis was performed using Python (pandas 2.x). Continuous variables with approximately normal distribution are presented as mean ± standard deviation (SD), and non-normally distributed variables as median with IQR. Categorical variables are presented as absolute frequencies and percentages. Pearson correlation was used to assess associations between echocardiographic and biochemical parameters. Due to the small sample size, multivariable analysis was not performed. Missing data were handled using complete-case analysis; denominators reflect patients with available data. NT-proBNP was available in 17/26 (65.4%). Results are hypothesis-generating.

## Results

### Demographic characteristics

**Figure 1:**
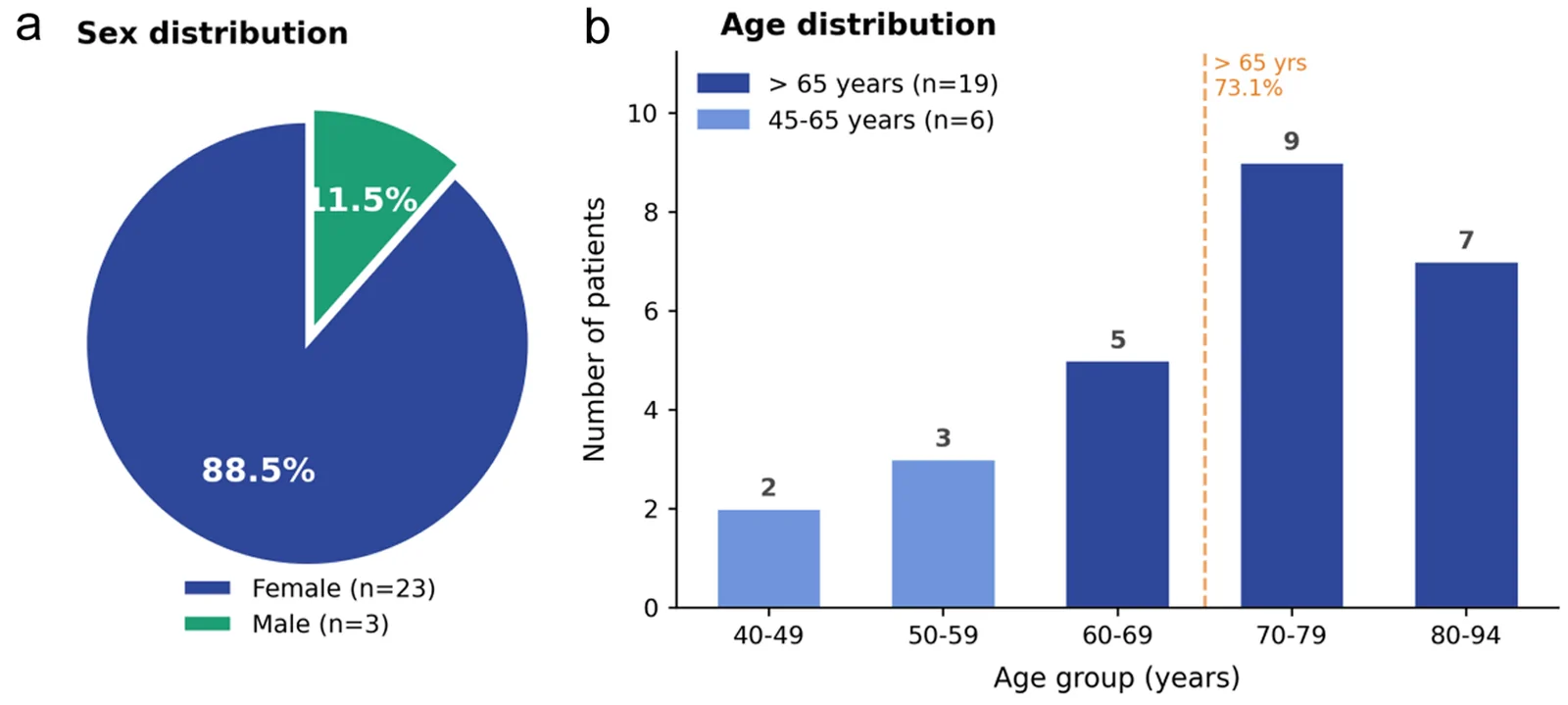
Demographic characteristics of the cohort. (a) Sex distribution: 88.5% women (n = 23) and 11.5% men (n = 3). (b) Age distribution: predominance of patients aged > 65 years (73.1%, n = 19).

The cohort comprised 23 women (88.5%) and three men (11.5%), with a female-to-male ratio of 7.7:1 [15]. Mean age was 71.4 ± 12.0 years (median 73.5; interquartile range (IQR) 66–80; range 44–91). By age group: one patient (3.8%) was < 45 years, six (23.1%) were 45–65 years, and 19 (73.1%) were > 65 years (Fig. 1).

### Triggering factors

**Figure 2:**
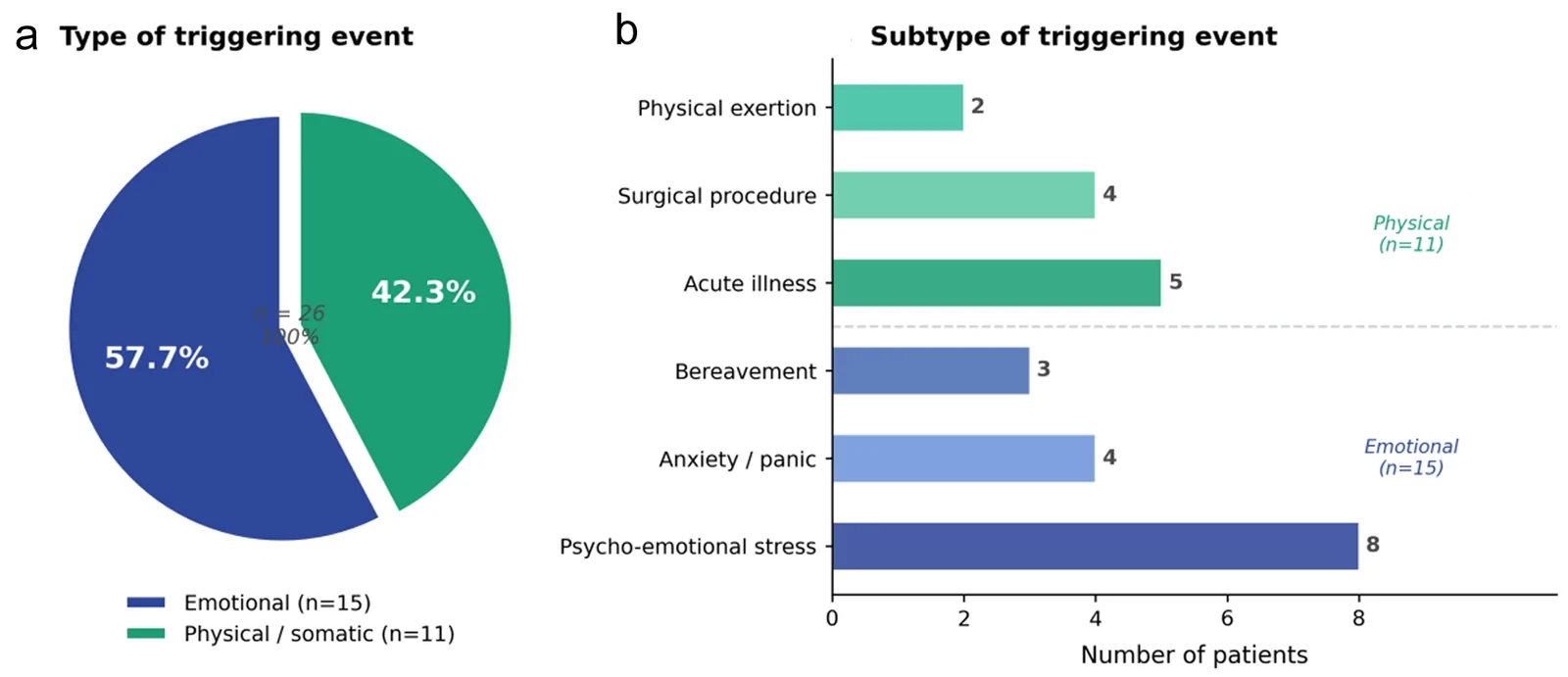
Triggering event. (a) Proportion of emotional vs. physical/somatic triggers. (b) Subcategory breakdown of trigger types.

A triggering event was identified in all 26 patients (100%). Emotional stressors accounted for 57.7% and physical/somatic triggers for 42.3% (Fig. 2).

### Clinical presentation

#### Symptoms and hemodynamics

Primary symptoms included chest pain (76.9%) and dyspnea (53.8%) [16], with co-occurrence of 30.8%. Syncope was reported in 7.7%. Mean systolic blood pressure (SBP) was 126 ± 19 mm Hg, diastolic blood pressure (DBP) was 74 ± 12 mm Hg, and heart rate (HR) was 88 ± 15 bpm.

#### Heart failure markers

Central venous congestion was reported in 17/25 (68%), and peripheral edema in 3/25 (12%). NYHA/Killip class ≥ 3 was identified in > 80% of assessed patients (Table 1).

**Table 1:** Baseline Clinical and Hemodynamic Characteristics

Parameter	Value (mean ± SD) or n (%)
Number of patients	26
Systolic BP (mm Hg)	126 ± 19
Diastolic BP (mm Hg)	74 ±12
Heart rate (bpm)	88 ± 15
Central venous congestion, n/N (%)	17/25 (68%)
Peripheral edema, n/N (%)	3/25 (12%)

BP: blood pressure; bpm: beats per minute; SD: standard deviation.

### ECG alterations

Admission ECG findings comprised isolated negative T-waves (five patients), ST-elevation, ST-depression, QRS changes (four each), and combinations in remainder. Dynamic evolution included negative T-waves in 62.5% and QTc prolongation in four patients [17, 18].

### Echocardiographic parameters

#### Systolic function

**Figure 3:**
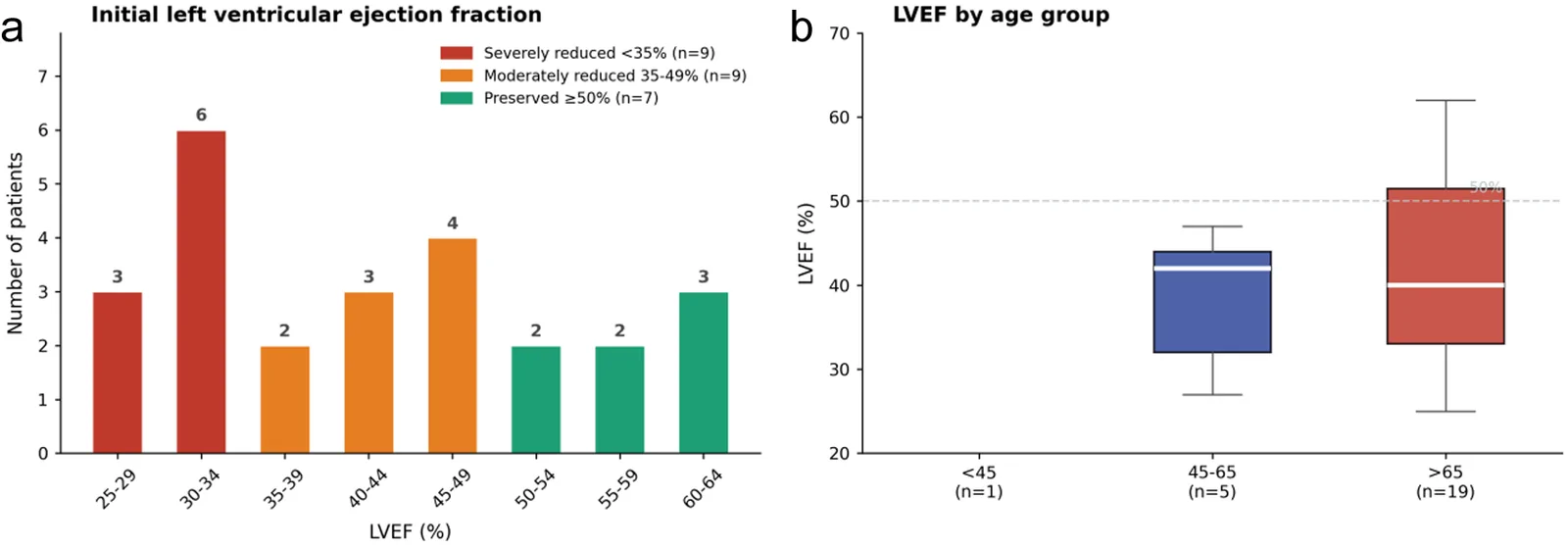
Initial LVEF. (a) Histogram color-coded by dysfunction severity: severely reduced < 35% (n = 9), moderately reduced 35–49% (n = 10), preserved ≥ 50% (n = 7). (b) LVEF by age group (boxplots). LVEF: left ventricular ejection fraction.

Echocardiography was performed in all 26 patients (100%). Classic apical ballooning was observed in 100% [19]. Mean LVEF was 41.8±11.4% (median 41%; IQR 33–50%). Severely reduced LVEF (< 35%) occurred in nine patients (34.6%), moderately reduced (35–49%) in 10 (38.5%), and preserved (≥ 50%) in seven (26.9%). Mean LVEDV was 104 ± 45 mL, and mean LVESV was 59.8 ± 32.4 mL (Fig. 3).

#### Adjunctive findings

**Figure 4:**
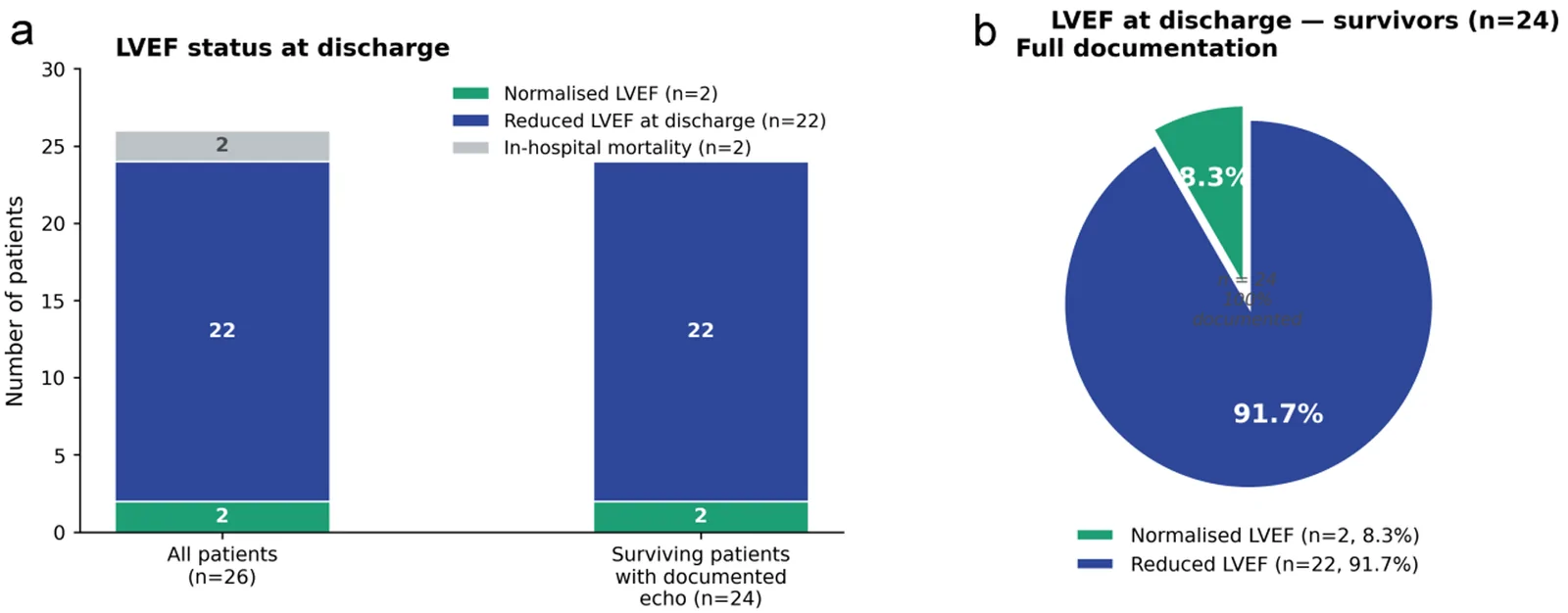
Control echocardiography at discharge. (a) All 26 patients. (b) 24 survivors, 91.7% with persistently reduced LVEF. LVEF: left ventricular ejection fraction.

Echocardiographically estimated pulmonary hypertension (PAH, defined as SPAP > 35 mm Hg) was present in 15/26 (57.7%). Valvular regurgitation was present in 6/25 (24%). LVOT obstruction was present in 1/21 (4.8%) (Fig. 4).

#### Discharge echocardiography

**Figure 5:**
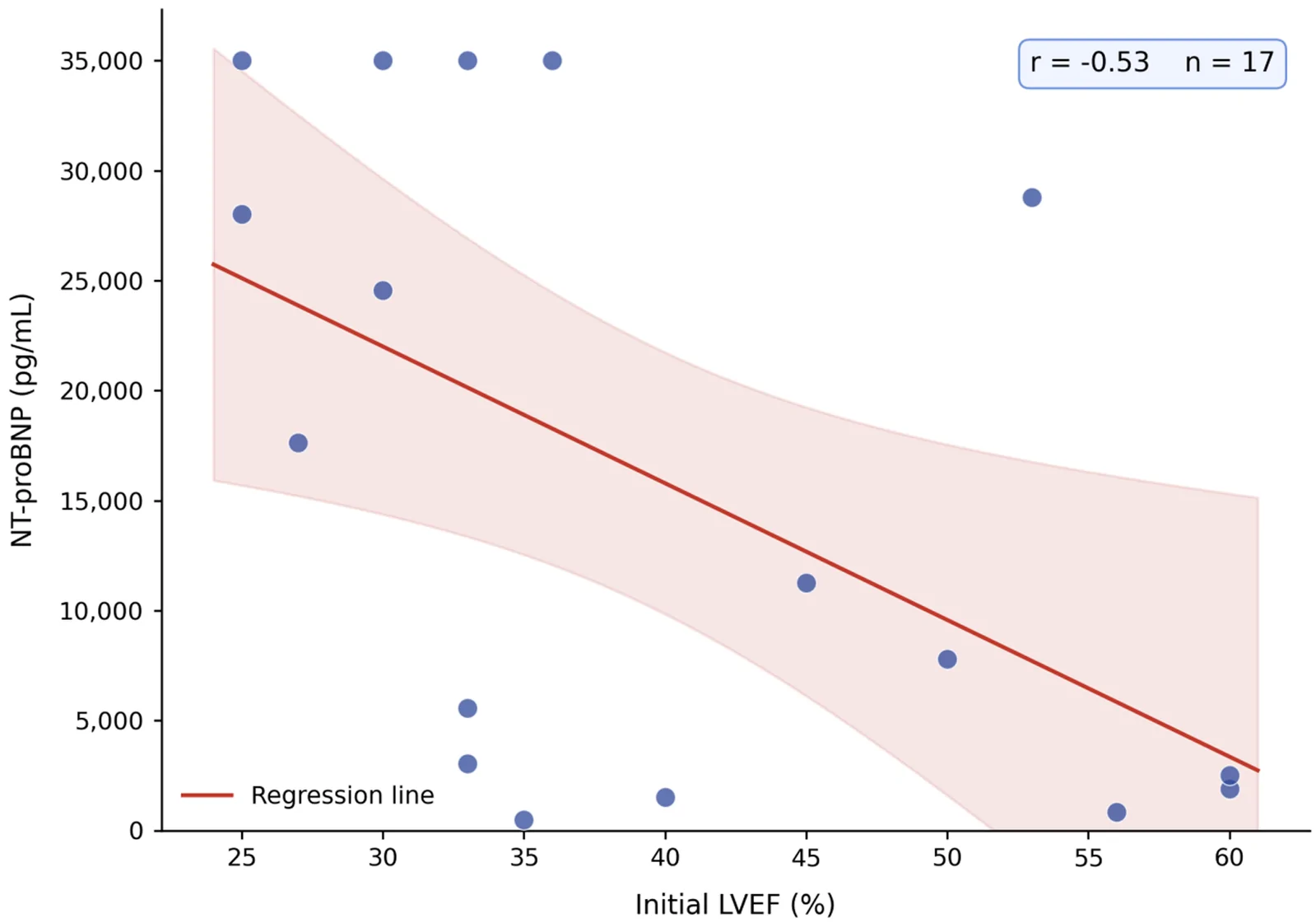
Scatter plot depicting the correlation between initial LVEF and NT-proBNP (n = 17), with linear regression line and 95% confidence interval. Pearson r = −0.53. LVEF: left ventricular ejection fraction; NT-proBNP: N-terminal pro-B-type natriuretic peptide.

Follow-up echocardiography was performed in all 24 surviving patients (100%) at a mean of 10 days after admission. LVEF remained reduced in 22/24 (91.7%) at the time of discharge. Normalization was documented in only 2/24 (8.3%), supporting the recommendation for outpatient echocardiographic follow-up after discharge (Fig. 5; Table 2).

**Table 2:** Echocardiographic Parameters

Parameter	Mean ± SD	Median	IQR/range
Baseline LVEF (%)	41.8 ± 11.4	41	33–50/25–62
LVEDV (mL)	104 ± 45	85	72–130
LVESV (mL)	59.8 ± 32.4	48	36–71
Apical ballooning, n/N (%)	26/26 (100%)	-	-
PAH, n/N (%)	15/26 (57.7%)	-	-
Valvular regurgitation, n/N (%)	6/25 (24%)	-	-
LVOT obstruction, n/N (%)	1/21 (4.8%)	-	-
LVEF recovery at discharge, n/N (%)	2/24 (8.3%)	-	-

IQR: interquartile range; LVEDV: LV end-diastolic volume; LVEF: LV ejection fraction; LVESV: LV end-systolic volume; LVOT: LV outflow tract; PAH: pulmonary arterial hypertension; SD: standard deviation.

### Laboratory parameters and biomarkers

Mean hs-TnT was 0.32 ± 0.40 ng/mL (median 0.16). Mean NT-proBNP (n = 17) was 16,116 ± 14,260 pg/mL (median 11,273), exceeding 10,000 pg/mL in > 50% of measured cases. Mean CRP was 34.6 ± 50.7 mg/L (median 13.0). Mean creatinine was 98.5 ± 40.2 µmol/L. Estimated glomerular filtration rate (eGFR) was 61.6 ± 26.5 mL/min/1.73 m^2^.

**Figure 6:**
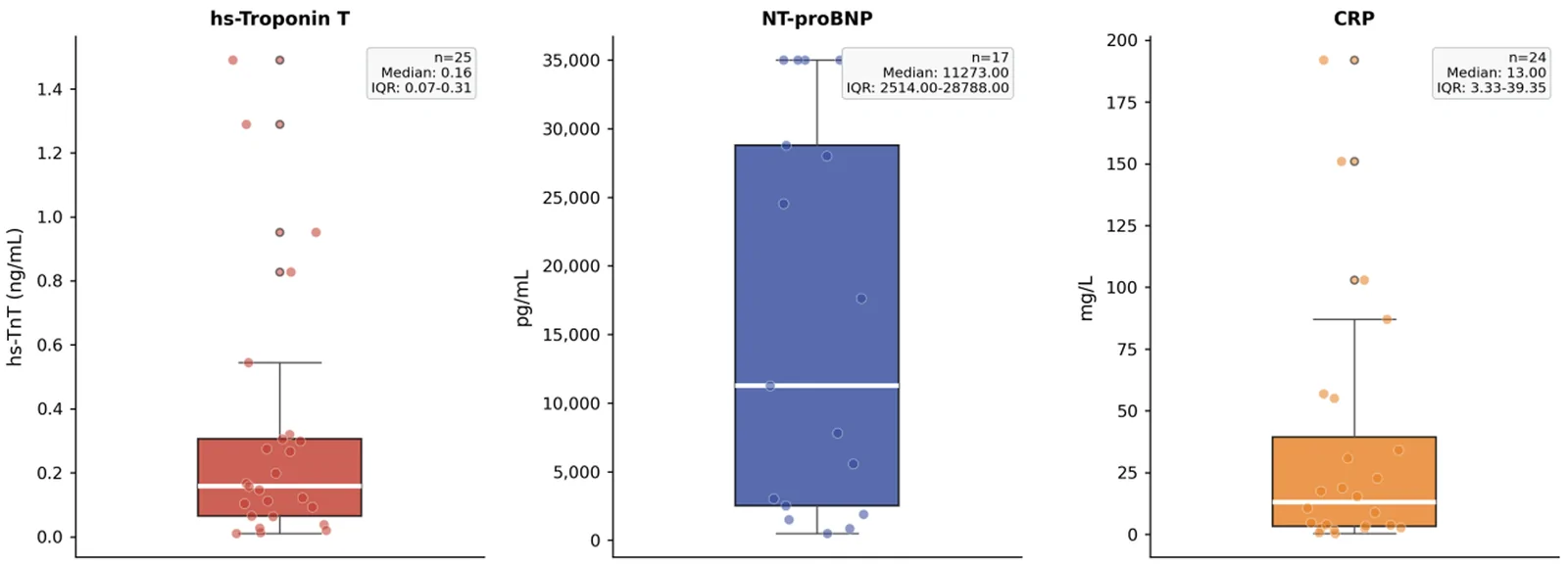
Cardiac biomarkers on admission. Box-strip plots for hs-TnT (ng/mL), NT-proBNP (pg/mL), and CRP (mg/L) with individual patient values overlaid. CRP: C-reactive protein; hs-TnT: high-sensitivity troponin T; NT-proBNP: N-terminal pro-B-type natriuretic peptide.

**Figure 7:**
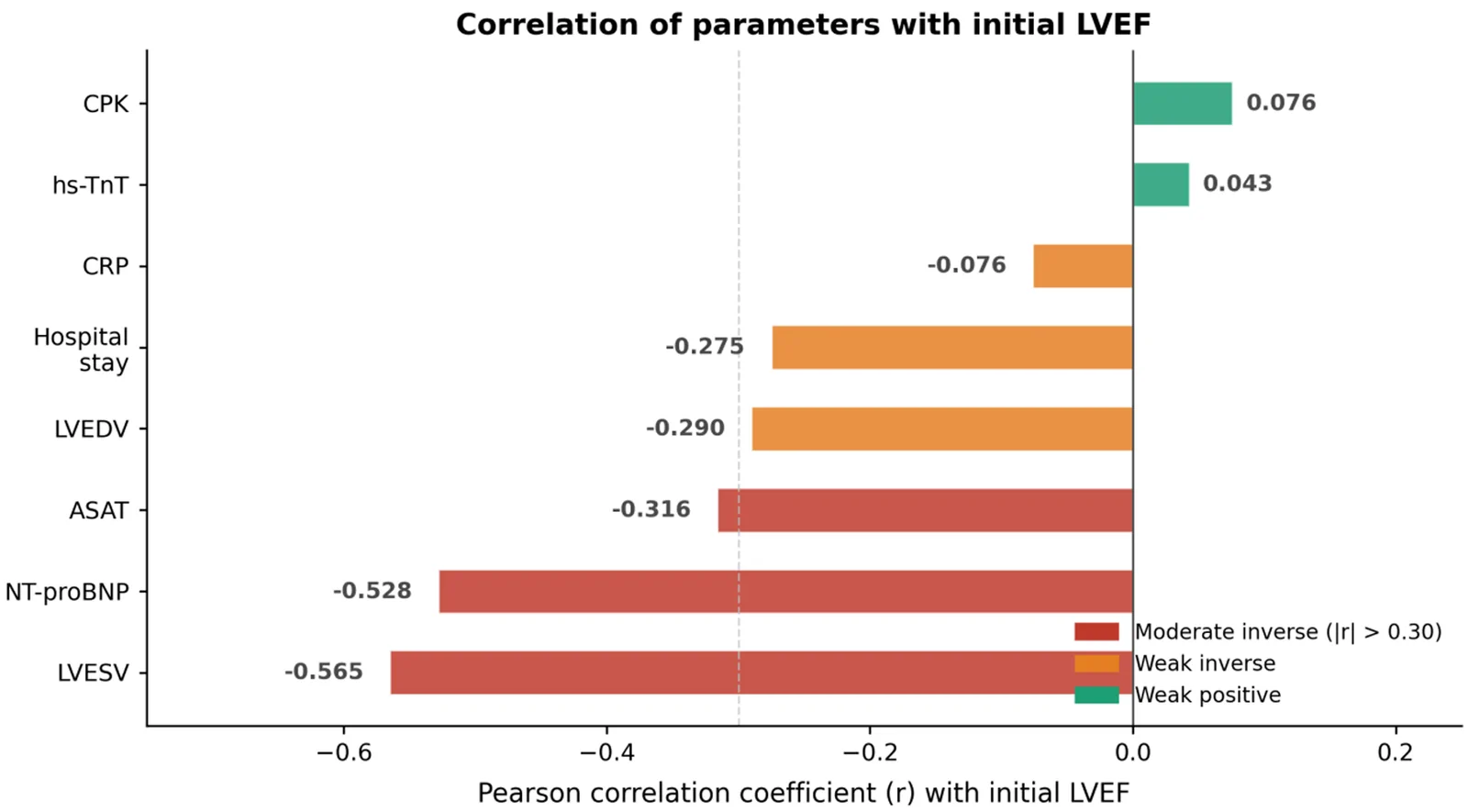
Pearson correlation coefficients (r) of clinical and biochemical parameters with initial LVEF. Red bars indicate a moderate inverse correlation (|r| > 0.30). LVEF: left ventricular ejection fraction.

**Figure 8:**
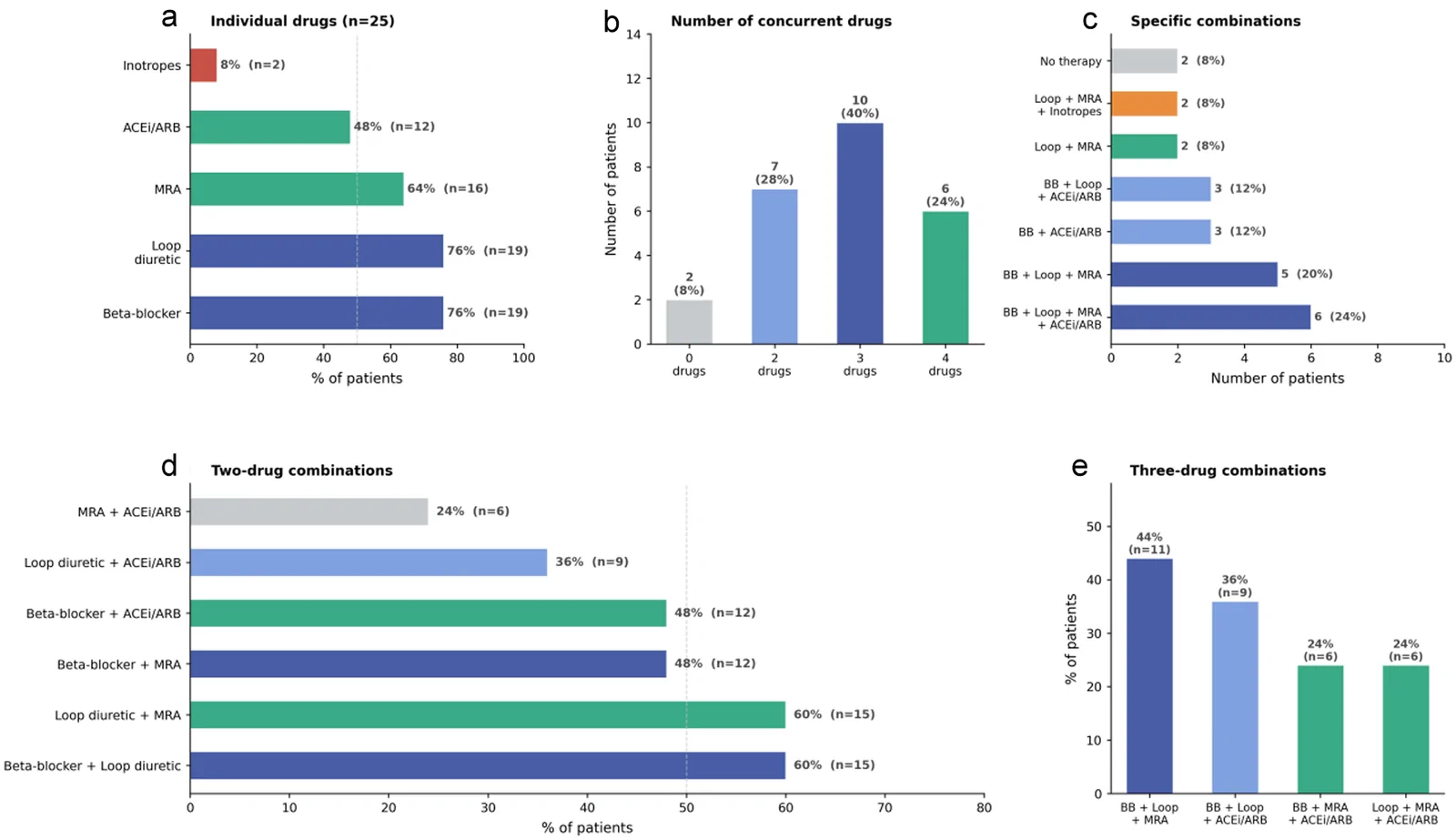
Pharmacological treatment. (a) Individual drug frequencies. (b) Number of concurrent drugs. (c) Specific regimens. (d) Two-drug combinations. (e) Three-drug combinations.

NT-proBNP showed a moderate inverse correlation with initial LVEF (ρ = −0.53, n = 17, Spearman). LVESV yielded a correlation coefficient of r = −0.57. Aspartate aminotransferase (AST) presented r = −0.32. Hs-TnT and CRP did not show significant correlation with LVEF (Figs. 6–8; Table 3).

**Table 3:** Biomarkers on Admission

Parameter	n	Mean ± SD	Median (IQR)
hs-TnT (ng/mL)	25	0.32 ± 0.40	0.16 (0.07–0.31)
NT-proBNP (pg/mL)	17	16,116 ± 14,260	11,273 (2,514–28,788)
CPK (U/L)	25	276.8 ± 426.3	135 (77–293)
CPK-MB (U/L)	25	42.6 ± 47.6	24 (17–46)
AST (U/L)	25	168.6 ± 449.5	42 (23–66)
CRP (mg/L)	24	34.6 ± 50.7	13.0 (3.3–39.4)
Creatinine (µmol/L)	25	98.5 ± 40.2	96 (74–116)
eGFR CKD-EPI (mL/min/1.73 m^2^)	25	61.6 ± 26.5	57 (42–73)
Hemoglobin (g/L)	25	128.5 ± 22.5	132 (117–140)

AST: aspartate aminotransferase; CKD-EPI: Chronic Kidney Disease Epidemiology Collaboration; CPK: creatine phosphokinase; CRP: C-reactive protein; eGFR: estimated GFR; hs-TnT: high-sensitivity troponin T; IQR: interquartile range; NT-proBNP: N-terminal pro-B-type natriuretic peptide.

### Coronary angiography

Coronary angiography was performed in all 26 patients. Significant obstructive CAD was present in four (15.4%), and absent in 22 (84.6%).

### Inpatient pharmacotherapy

**Figure 9:**
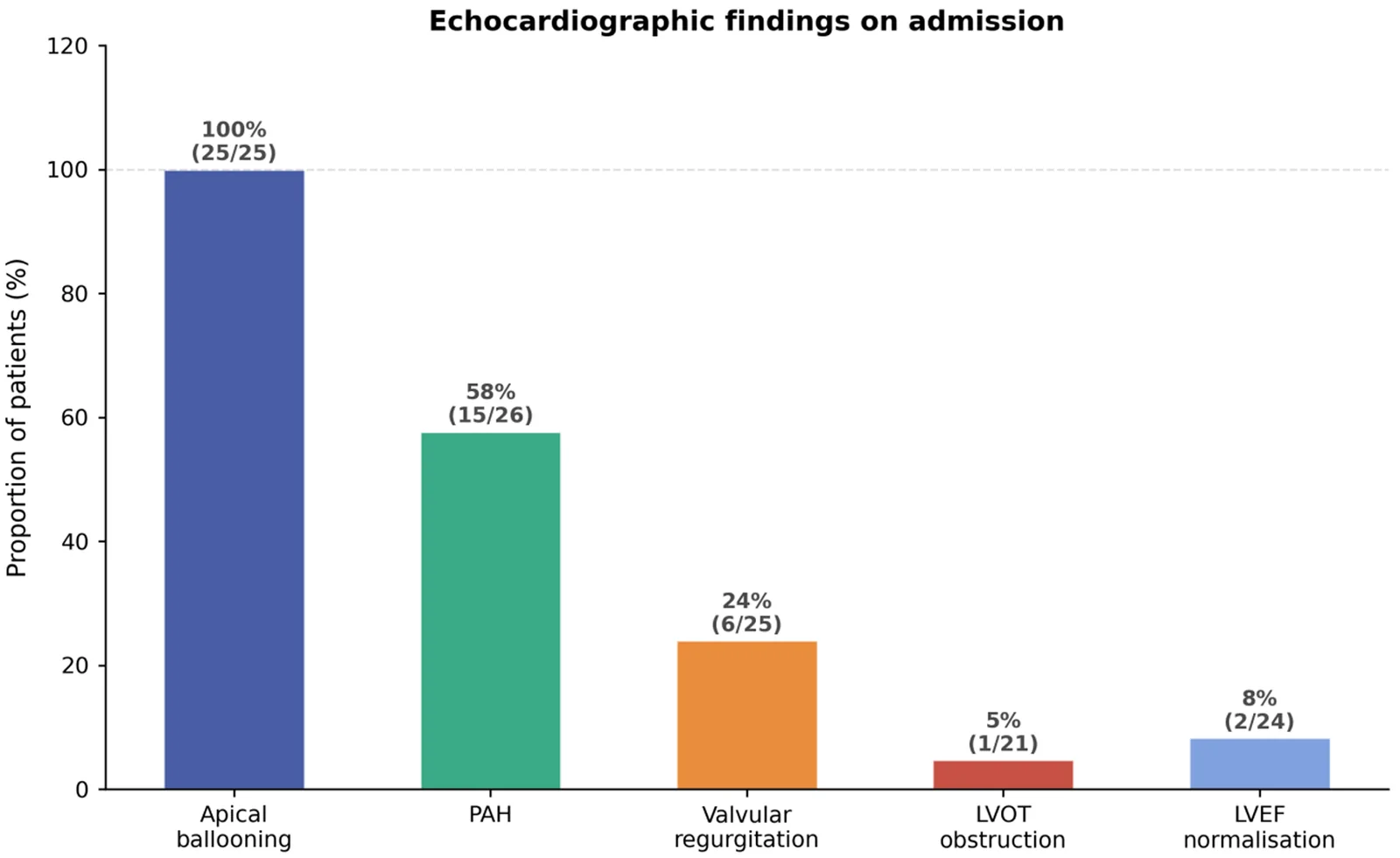
Echocardiographic findings on admission: apical ballooning (100%), PAH (57.7%), valvular regurgitation (24%), LVOT obstruction (4.8%), LVEF normalization at discharge (8.3%). LVEF: left ventricular ejection fraction; LVOT: LV outflow tract; PAH: pulmonary arterial hypertension.

Beta-blockers (BBs) were administered to 76% of patients, loop diuretics 76%, mineralocorticoid receptor antagonists (MRAs) 64%, angiotensin-converting enzyme inhibitor/angiotensin II receptor blocker (ACEi/ARB) 48%, and inotropes 8%. No patient received monotherapy. Most frequent regimen was BB + loop diuretic + MRA (44%), followed by quadruple therapy with ACEi/ARB (24%) (Fig. 9).

### Hospitalization and outcomes

**Figure 10:**
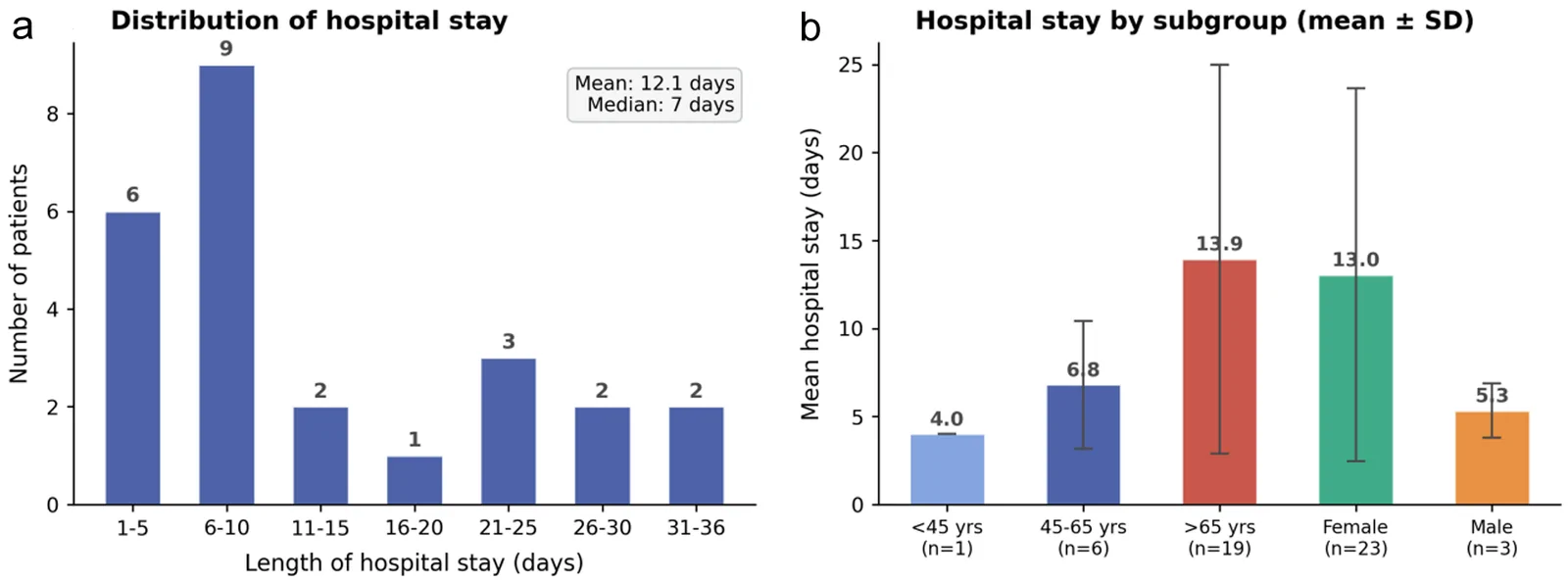
LOS. (a) Distribution across the full cohort. (b) Mean LOS (± SD) stratified by age group and sex. LOS: length of hospital stay; SD: standard deviation.

Mean LOS was 12.1 ± 10.2 days (median 7; range 1–36). LOS was longer in patients > 65 years (13.9 vs. 6.8 days) and women (13.0 vs. 5.3 days). In-hospital complications occurred in 5/20 survivors (25%), all female: atrial fibrillation (n = 3) and ventricular ectopy (Lown class II, n = 2) [20]. In-hospital mortality was 7.7% (2/26), both women > 65 years (Fig. 10; Table 4).

**Table 4:** Hospitalization Duration and Outcomes

Parameter	Value
Mean LOS (days), mean ± SD	12.1 ± 10.2
Median LOS (days), IQR	7 (5–20)
LOS > 65 years (days), mean ± SD	13.9 ± 11.0
LOS 45–65 years (days), mean ± SD	6.8 ± 3.6
LOS females (days), mean ± SD	13.0 ± 10.6
LOS males (days), mean ± SD	5.3 ± 1.5
In-hospital complications, n/N (%)	5/20 (25.0%)
In-hospital mortality, n (%)	2/26 (7.7%)

LOS: length of hospital stay; SD: standard deviation.

## Discussion

This single-center retrospective study describes the clinical and diagnostic characteristics of 26 consecutively hospitalized patients with TTS over a 5-year period. To our knowledge, this represents one of the first systematically documented TTS case series from Bulgaria, contributing clinical data from a Central and Eastern European population that remains substantially underrepresented in large international registries [21]. The female predominance (88.5%, ratio 7.7:1) is fully consistent with the InterTAK registry (89.8% women, mean age 67 years) [6]. The mean age in our cohort (71.4 ± 12.0 years) is slightly higher than InterTAK, which may account for the more severe clinical presentation. The GEIST registry confirmed that age ≥ 75 years and LVEF ≤ 35% are independent predictors of in-hospital mortality [22]; both deceased patients in our cohort were > 65 years with initial LVEF ≤ 50%.

A triggering event was identified in 100% of our patients (57.7% emotional, 42.3% physical), compared to ∼70% in InterTAK [6]. The temporal shift towards physical triggers (39% to 58%, 2004–2021) documented by Schweiger et al is prognostically significant, as physically triggered TTS is associated with higher mortality [10, 22].

The mean initial LVEF (41.8±11.4%) was virtually identical to InterTAK (40.7±11.2%) [6]. The ESC Expert Consensus states that LV contractility typically recovers within 4–8 weeks [13], explaining the low normalization rate at discharge (8.3%) and supporting mandatory outpatient echocardiographic follow-up. The relatively high PAH rate (57.7%) may reflect the advanced mean age and hemodynamic compromise in this cohort.

The moderate inverse correlation between NT-proBNP and LVEF (ρ = −0.53, Spearman) is consistent with the ESC consensus [13]. The disproportionate BNP/TnT elevation characteristic of TTS [23] was also observed. A recent ATAK registry analysis demonstrated that CRP > 33 mg/L at discharge predicts 1-year mortality (AUC = 0.81) [24], a threshold exceeded by ∼40% of our patients.

In-hospital mortality of 7.7% is at the upper limit of published rates (1–8%) [25]. CAD was found in 15.4%—identical to InterTAK’s 15.3% [6]. Pharmacological management was heterogeneous, consistent with the absence of prospective randomized data for TTS-specific therapy [13]. BB use (76%) was comparable to InterTAK at discharge (78.1%) [6]. A recent meta-analysis showed no significant reduction in TTS recurrence with BBs or ACEi/ARB [26, 27].

### Limitations

This study has several important limitations. First, the small sample size (n = 26) substantially limits statistical power, and all correlation analyses should be considered exploratory only. Second, the retrospective, single-center design introduces potential selection bias. Third, the absence of a control group precludes direct comparison with ACS or general hospitalized populations. Fourth, myocarditis was excluded on clinical and echocardiographic grounds without routine cardiac magnetic resonance imaging. Fifth, NT-proBNP data were available in only 65.4% of patients. Sixth, there are no long-term follow-up data beyond the index hospitalization. Despite these limitations, this study provides systematic documentation of a consecutive Bulgarian cohort with comprehensive clinical, biochemical, and echocardiographic characterization.

### Conclusions

This single-center retrospective observational study is consistent with the major epidemiological and clinical characteristics of TTS described in large international registries: predominance in elderly postmenopausal women, universal triggering events, significant systolic dysfunction at presentation (mean LVEF 41.8%), and a moderate inverse correlation between NT-proBNP and initial LVEF consistent with international registry data.

In-hospital mortality (7.7%) and complication rates (25% of survivors) are in the upper range of published values, reflecting the advanced mean age of this cohort. LVEF remained reduced at discharge in 91.7% of surviving patients, underscoring the necessity of mandatory outpatient echocardiographic surveillance.

These hypothesis-generating findings should be validated in larger prospective multicenter cohorts. Furthermore, prospective randomized studies evaluating different pharmacological treatment strategies with long-term follow-up are needed to guide evidence-based management of TTS. The present data contribute to the local epidemiology of TTS and may serve as the foundation for a future national multicenter registry.

## Data Availability

The data that support the findings of this study are available from the corresponding author upon reasonable request.

## Abbreviations

ACS: acute coronary syndrome
CAD: coronary artery disease
CPK: creatine phosphokinase
CRP: C-reactive protein
DBP: diastolic blood pressure
ECG: electrocardiogram
eGFR: estimated glomerular filtration rate
HR: heart rate
hs-TnT: high-sensitivity troponin T
IQR: interquartile range
LOS: length of hospital stay
LV: left ventricle/ventricular
LVEDV: LV end-diastolic volume
LVEF: LV ejection fraction
LVESV: LV end-systolic volume
LVOT: LV outflow tract
MRA: mineralocorticoid receptor antagonists
NT-proBNP: N-terminal pro-B-type natriuretic peptide
NYHA: New York Heart Association
PAH: pulmonary arterial hypertension
SBP: systolic blood pressure
SD: standard deviation
TTS: Takotsubo syndrome
